# Effectiveness of Maternal Vaccination with mRNA COVID-19 Vaccine During Pregnancy Against COVID-19–Associated Hospitalization in Infants Aged <6 Months — 17 States, July 2021–January 2022

**DOI:** 10.15585/mmwr.mm7107e3

**Published:** 2022-02-18

**Authors:** Natasha B. Halasa, Samantha M. Olson, Mary A. Staat, Margaret M. Newhams, Ashley M. Price, Julie A. Boom, Leila C. Sahni, Melissa A. Cameron, Pia S. Pannaraj, Katherine E. Bline, Samina S. Bhumbra, Tamara T. Bradford, Kathleen Chiotos, Bria M. Coates, Melissa L. Cullimore, Natalie Z. Cvijanovich, Heidi R. Flori, Shira J. Gertz, Sabrina M. Heidemann, Charlotte V. Hobbs, Janet R. Hume, Katherine Irby, Satoshi Kamidani, Michele Kong, Emily R. Levy, Elizabeth H. Mack, Aline B. Maddux, Kelly N. Michelson, Ryan A. Nofziger, Jennifer E. Schuster, Stephanie P. Schwartz, Laura Smallcomb, Keiko M. Tarquinio, Tracie C. Walker, Matt S. Zinter, Suzanne M. Gilboa, Kara N. Polen, Angela P. Campbell, Adrienne G. Randolph, Manish M. Patel, Overcoming COVID-19 Investigators, Overcoming COVID-19 Network, Laura D. Zambrano, Meghan Murdock, Mary Glas Gaspers, Connor P. Kelley, Katri V. Typpo, Peter M. Mourani, Ronald C. Sanders, Chelsea Smith, Masson Yates, Katheryn Crane, Geraldina Lionetti, Juliana Murcia-Montoya, Denise Villarreal-Chico, Daniel Hakimi, Adam L. Skura, Imogene Carson, Justin M. Lockwood, Emily Port, Brandon M. Chatani, Nadine Baida, Laila Hussaini, Hassan A. Khan, Simone T. Rhodes, Courtney M. Rowan, Mary Stumpf, Marla S. Johnston, Laura Berbert, Benjamin J. Boutselis, Sabrina R. Chen, Jie He, Suden Kucukak, Timothy P. McCadden, Amber O. Orzel, Edie Weller, Patrick Moran, Ellen R. Bruno, Lexie A. Goertzen, Supriya Behl, Noelle M. Drapeau, Lacy Malloch, Lora Martin, April Palmer, Roberto P. Santos, Abigail Kietzman, Melissa Sullivan, Lauren A. Hoody, Valerie H. Rinehart, Paris C. Bennett, Merry L. Tomcany, Nicole A. Twinem, Chelsea C. Rohlfs, Amber Wolfe, Rebecca L. Douglas, Kathlyn Phengchomphet, Megan M. Bickford, Lauren E. Wakefield, Meena Golchha, Laura S. Stewart, Jennifer N. Oates, Cindy Bowens, Mia Maamari

**Affiliations:** ^1^Division of Pediatric Infectious Diseases, Department of Pediatrics, Vanderbilt University Medical Center, Nashville, Tennessee; ^2^CDC COVID-19 Emergency Response Team; ^3^Department of Pediatrics, University of Cincinnati College of Medicine, Cincinnati Children’s Hospital Medical Center, Cincinnati, Ohio; ^4^Department of Anesthesiology, Critical Care, and Pain Medicine, Boston Children’s Hospital, Boston, Massachusetts; ^5^Department of Pediatrics, Baylor College of Medicine, Immunization Project, Texas Children’s Hospital, Houston, Texas; ^6^Division of Pediatric Hospital Medicine, UC San Diego-Rady Children’s Hospital, San Diego, California; ^7^Division of Infectious Diseases, Children’s Hospital Los Angeles and Departments of Pediatrics and Molecular Microbiology and Immunology, University of Southern California, Los Angeles, California; ^8^Division of Pediatric Critical Care Medicine, Nationwide Children’s Hospital, Columbus, Ohio; ^9^The Ryan White Center for Pediatric Infectious Disease and Global Health, Department of Pediatrics, Indiana University School of Medicine, Indianapolis, Indiana; ^10^Department of Pediatrics, Division of Cardiology, Louisiana State University Health Sciences Center and Children’s Hospital of New Orleans, New Orleans, Louisiana; ^11^Division of Critical Care Medicine, Department of Anesthesiology and Critical Care, Children’s Hospital of Philadelphia, Philadelphia, Pennsylvania; ^12^Division of Critical Care Medicine, Department of Pediatrics, Northwestern University Feinberg School of Medicine, Ann & Robert H. Lurie Children’s Hospital of Chicago, Chicago, Illinois; ^13^Division of Pediatric Critical Care, Department of Pediatrics, Children’s Hospital and Medical Center, Omaha, Nebraska; ^14^Division of Critical Care Medicine, UCSF Benioff Children’s Hospital Oakland, California; ^15^Division of Pediatric Critical Care Medicine, Department of Pediatrics, Mott Children’s Hospital and University of Michigan, Ann Arbor, Michigan; ^16^Division of Pediatric Critical Care, Department of Pediatrics, Cooperman Barnabas Medical Center, Livingston, New Jersey; ^17^Division of Pediatric Critical Care Medicine, Children’s Hospital of Michigan, Central Michigan University, Detroit, Michigan; ^18^Department of Pediatrics, Department of Microbiology, Division of Infectious Diseases, University of Mississippi Medical Center, Jackson, Mississippi; ^19^Division of Pediatric Critical Care, University of Minnesota Masonic Children’s Hospital, Minneapolis, Minnesota; ^20^Section of Pediatric Critical Care, Department of Pediatrics, Arkansas Children’s Hospital, Little Rock, Arkansas; ^21^The Center for Childhood Infections and Vaccines of Children’s Healthcare of Atlanta and the Department of Pediatrics, Emory University School of Medicine; Atlanta, Georgia; ^22^Division of Pediatric Critical Care Medicine, Department of Pediatrics, University of Alabama at Birmingham, Birmingham, Alabama; ^23^Divisions of Pediatric Infectious Diseases and Pediatric Critical Care Medicine, Department of Pediatric and Adolescent Medicine, Mayo Clinic, Rochester, Minnesota; ^24^Division of Pediatric Critical Care Medicine, Medical University of South Carolina, Charleston, South Carolina; ^25^Department of Pediatrics, Section of Critical Care Medicine, University of Colorado School of Medicine and Children’s Hospital Colorado, Aurora, Colorado; ^26^Division of Critical Care Medicine, Department of Pediatrics, Akron Children’s Hospital, Akron, Ohio; ^27^Division of Pediatric Infectious Diseases, Department of Pediatrics, Children’s Mercy Kansas City, Kansas City, Missouri; ^28^Department of Pediatrics, University of North Carolina at Chapel Hill Children’s Hospital, Chapel Hill, North Carolina; ^29^Department of Pediatrics, Medical University of South Carolina, Charleston, South Carolina; ^30^Division of Critical Care Medicine, Department of Pediatrics, Emory University School of Medicine, Children’s Healthcare of Atlanta, Atlanta, Georgia; ^31^Department of Pediatrics, Divisions of Critical Care Medicine and Allergy, Immunology, and Bone Marrow Transplant, University of California San Francisco, San Francisco, California; ^32^Departments of Anaesthesia and Pediatrics, Harvard Medical School, Boston, Massachusetts.; CDC; Children’s of Alabama, Birmingham, Alabama; University of Arizona, Tucson, Arizona; University of Arizona, Tucson, Arizona; University of Arizona, Tucson, Arizona; Arkansas Children’s Hospital, Little Rock, Arkansas; Arkansas Children’s Hospital, Little Rock, Arkansas; Arkansas Children’s Hospital, Little Rock, Arkansas; Arkansas Children’s Hospital, Little Rock, Arkansas; Rady Children’s Hospital, San Diego, California; University of California; San Francisco Benioff Children’s Hospital Oakland, Oakland, California; University of California; San Francisco Benioff Children’s Hospital Oakland, Oakland, California; University of California San Francisco Benioff Children’s Hospital, San Francisco, California; Children’s Hospital Los Angeles, Los Angeles, California; Children’s Hospital Los Angeles, Los Angeles, California; Children’s Hospital Colorado, Aurora, Colorado; Children’s Hospital Colorado, Aurora, Colorado; Children’s Hospital Colorado, Aurora, Colorado; Holtz Children’s Hospital, Miami, Florida; Emory University School of Medicine and Children’s Healthcare of Atlanta, Atlanta, Georgia; Emory University School of Medicine and Children’s Healthcare of Atlanta, Atlanta, Georgia; Ann & Robert H. Lurie Children’s Hospital of Chicago, Chicago, Illinois; Ann & Robert H. Lurie Children’s Hospital of Chicago, Chicago, Illinois; Riley Hospital for Children, Indianapolis, Indiana; Riley Hospital for Children, Indianapolis, Indiana; Children’s Hospital of New Orleans, New Orleans, Louisiana; Boston Children’s Hospital, Boston, Massachusetts; Boston Children’s Hospital, Boston, Massachusetts; Boston Children’s Hospital, Boston, Massachusetts; Boston Children’s Hospital, Boston, Massachusetts; Boston Children’s Hospital, Boston, Massachusetts; Boston Children’s Hospital, Boston, Massachusetts; Boston Children’s Hospital, Boston, Massachusetts; Boston Children’s Hospital, Boston, Massachusetts; University of Michigan CS Mott Children’s Hospital, Ann Arbor, Michigan; University of Minnesota Masonic Children’s Hospital, Minneapolis, Minnesota; University of Minnesota Masonic Children’s Hospital, Minneapolis, Minnesota; Mayo Clinic, Rochester, Minnesota; Mayo Clinic, Rochester, Minnesota; Children’s Hospital of Mississippi, Jackson, Mississippi; Children’s Hospital of Mississippi, Jackson, Mississippi; Children’s Hospital of Mississippi, Jackson, Mississippi; Children’s Hospital of Mississippi, Jackson, Mississippi; Children’s Mercy Kansas City, Kansas City, Missouri; Children’s Mercy Kansas City, Kansas City, Missouri; Children’s Hospital & Medical Center, Omaha, Nebraska; Children’s Hospital & Medical Center, Omaha, Nebraska; University of North Carolina at Chapel Hill, Chapel Hill, North Carolina; Akron Children’s Hospital, Akron, Ohio; Akron Children’s Hospital, Akron, Ohio; Cincinnati Children’s Hospital, Cincinnati, Ohio; Nationwide Children’s Hospital, Columbus, Ohio; Children’s Hospital of Philadelphia, Philadelphia, Pennsylvania; Children’s Hospital of Philadelphia, Philadelphia, Pennsylvania; Medical University of South Carolina Children’s Health, Charleston, South Carolina; Medical University of South Carolina Children’s Health, Charleston, South Carolina; Monroe Carell Jr. Children’s Hospital at Vanderbilt, Nashville, Tennessee; Monroe Carell Jr. Children’s Hospital at Vanderbilt, Nashville, Tennessee; Texas Children’s Hospital and Baylor College of Medicine, Houston, Texas; University of Texas Southwestern, Children’s Medical Center Dallas, Dallas, Texas; University of Texas Southwestern, Children’s Medical Center Dallas, Dallas, Texas

COVID-19 vaccination is recommended for persons who are pregnant, breastfeeding, trying to get pregnant now, or who might become pregnant in the future, to protect them from COVID-19.^§^ Infants are at risk for life-threatening complications from COVID-19, including acute respiratory failure (*1*). Evidence from other vaccine-preventable diseases suggests that maternal immunization can provide protection to infants, especially during the high-risk first 6 months of life, through passive transplacental antibody transfer (*2*). Recent studies of COVID-19 vaccination during pregnancy suggest the possibility of transplacental transfer of SARS-CoV-2–specific antibodies that might provide protection to infants (*3*–*5*); however, no epidemiologic evidence currently exists for the protective benefits of maternal immunization during pregnancy against COVID-19 in infants. The Overcoming COVID-19 network conducted a test-negative, case-control study at 20 pediatric hospitals in 17 states during July 1, 2021–January 17, 2022, to assess effectiveness of maternal completion of a 2-dose primary mRNA COVID-19 vaccination series during pregnancy against COVID-19 hospitalization in infants. Among 379 hospitalized infants aged <6 months (176 with COVID-19 [case-infants] and 203 without COVID-19 [control-infants]), the median age was 2 months, 21% had at least one underlying medical condition, and 22% of case- and control-infants were born premature (<37 weeks gestation). Effectiveness of maternal vaccination during pregnancy against COVID-19 hospitalization in infants aged <6 months was 61% (95% CI = 31%–78%). Completion of a 2-dose mRNA COVID-19 vaccination series during pregnancy might help prevent COVID-19 hospitalization among infants aged <6 months.

Using a test-negative, case-control study design, vaccine performance was assessed by comparing the odds of having completed a 2-dose primary mRNA COVID-19 vaccination series during pregnancy among mothers of case-infants and control-infants (those with negative SARS-CoV-2 test results) (*6*). Participating infants were aged <6 months and admitted outside of their birth hospitalization to one of 20 pediatric hospitals during July 1, 2021–January 17, 2022. During this period, the SARS-CoV-2 Delta variant was the predominant variant in the United States through mid-December, after which Omicron became predominant.^¶^ Case-infants were hospitalized with COVID-19 as the primary reason for admission or had clinical symptoms consistent with acute COVID-19,** and case-infants had a positive SARS-CoV-2 reverse transcription–polymerase chain reaction (RT-PCR) or antigen test result. No case-infant received a diagnosis of multisystem inflammatory syndrome. Control-infants were those hospitalized with or without COVID-19 symptoms and with negative SARS-CoV-2 RT-PCR or antigen test results. Enrolled control-infants were matched to case-infants by site and were hospitalized within 3–4 weeks of a case-infant’s admission date. Baseline demographic characteristics, clinical information, and SARS-CoV-2 testing history were obtained through parent or guardian interviews performed by trained study personnel during hospitalization or after discharge, and electronic medical record review of the infant’s record. Mothers were asked about their COVID-19 vaccination history, including number of doses and whether a dose had been received during pregnancy, location where vaccine was received, vaccine manufacturer, and availability of a COVID-19 vaccination card. Study personnel reviewed documented sources, including state vaccination registries, electronic medical records, or other sources (e.g., documentation from primary care providers) to verify vaccination status.

Mothers were considered vaccinated against COVID-19 if they completed a 2-dose series of either Pfizer-BioNTech or Moderna mRNA COVID-19 vaccine, based on source documentation or by plausible self-report (provision of vaccination dates and location). Maternal COVID-19 vaccination status was categorized as 1) unvaccinated (mothers who did not receive COVID-19 vaccine before their infants’ hospitalization) or 2) vaccinated^††^ (mothers who completed their 2-dose primary mRNA COVID-19 vaccine series during pregnancy ≥14 days before delivery). SARS-CoV-2 infection status of the mother during pregnancy or after delivery was not documented in this evaluation. Mothers were excluded if they were partially vaccinated during pregnancy (1 dose during pregnancy and none before pregnancy) or vaccinated after pregnancy (71), received Janssen (Johnson & Johnson) COVID-19 vaccine (four), received 2 doses of COVID-19 vaccination before pregnancy (seven), or received >2 doses of COVID-19 vaccine ≥14 days before delivery (10).

Descriptive statistics (Pearson chi-square tests and Fisher’s exact tests for categorical outcomes or Wilcoxon rank-sum tests for continuous outcomes) were used to compare characteristics of case- and control-infants; p-values <0.05 were considered statistically significant. Effectiveness of maternal vaccination (i.e., vaccine effectiveness [VE]) against infant COVID-19 hospitalization was calculated using the equation VE = 100% × (1 – adjusted odds ratio of completing 2-doses of COVID-19 mRNA vaccines during pregnancy among mothers of case-infants and control-infants), determined from logistic regression models. Models were adjusted for infant age and sex, U.S. Census region, calendar time of admission, and race/ethnicity (*6*). Other factors were assessed (e.g., infant’s underlying health conditions, Social Vulnerability Index, and behavioral factors) but were not included in the final model because they did not change the odds ratio of vaccination by >5% or because data on many infants were not available (e.g., breastfeeding history, prematurity, or child care attendance). In a secondary analysis, effectiveness of maternal receipt of the second dose of COVID-19 vaccination early in pregnancy (within the first 20 weeks) and late in pregnancy (21 weeks through 14 days before delivery) was assessed. Statistical analyses were conducted using SAS (version 9.4; SAS Institute). Procedures were approved as public health surveillance by each participating site and CDC and were conducted consistent with applicable federal law and CDC policy.^§§^

During July 1, 2021–January 17, 2022, among 483 eligible infants in 20 pediatric hospitals in 17 states, 104 (22%) were excluded; 71 excluded infants were born to mothers partially vaccinated during pregnancy or vaccinated after delivery, 10 were born to mothers who received a third vaccine dose ≥14 days before delivery, and 23 were excluded for other reasons.^¶¶^ Among the remaining 379 hospitalized infants (176 case-infants and 203 control-infants), the median age was 2 months, 80 (21%) had at least one underlying medical condition, and 72 (22%) were born premature (Table 1). Among case-infants, 16% of mothers had received 2 COVID-19 vaccine doses during pregnancy, whereas 32% of control-infant mothers were vaccinated. Case- and control-infants had similar prevalences of underlying medical conditions (20% and 23%, respectively; p = 0.42) and prematurity (23% and 21%, respectively; p = 0.58). Case-infants were more commonly non-Hispanic Black (18%) and Hispanic (34%) than were control-infants (9% and 28%, respectively).

**TABLE 1:** Characteristics of infants aged <6 months hospitalized with COVID-19 (case-infants) and without COVID-19 (control-infants) — 20 pediatric hospitals, 17 states,* July 2021–January 2022

Characteristic (no. missing)	Case status, n/N^†^ (column %)		p-value^§^
	Case-infants (N = 176)	Control-infants (N = 203)	
**Median age, mos (IQR)**	2 (1–3)	2 (1–3)	0.96
**Age group, mos**			
0–2	129 (73.3)	153 (75.4)	0.64
3–5	47 (26.7)	50 (24.6)	
**Sex**			
Female	84 (47.7)	83 (40.9)	0.18
**Race and ethnicity**			
Black, non-Hispanic	32 (18.2)	19 (9.4)	0.02
White, non-Hispanic	56 (31.8)	82 (40.4)	
Other, non-Hispanic	10 (5.7)	21 (10.3)	
Hispanic, any race	60 (34.1)	56 (27.6)	
Unknown	18 (10.2)	25 (12.3)	
**Social Vulnerability Index,**^¶^ **(IQR) (1)**	0.71 (0.39–0.86)	0.61 (0.29–0.83)	0.06
U.S. Census region*			
Northeast	30 (17.1)	29 (14.3)	0.08
Midwest	44 (25.0)	60 (29.6)	
South	54 (30.7)	42 (20.7)	
West	48 (27.3)	72 (35.5)	
**Month of admission**			
July	10 (5.7)	5 (2.5)	0.14
August	23 (13.1)	26 (12.8)	
September	16 (9.1)	25 (12.3)	
October	10 (5.7)	21 (10.3)	
November	18 (10.2)	30 (14.8)	
December	59 (33.5)	51 (25.1)	
January**	40 (22.7)	45 (22.2)	
**Underlying health condition in infants**			
At least one underlying condition (5)	34/174 (19.5)	46/200 (23.0)	0.42
Respiratory disorder (6)	9/174 (5.2)	9/199 (4.5)	0.77
Cardiovascular system disorder (5)	15/174 (8.6)	19/200 (9.5)	0.77
Neurologic/Neuromuscular disorder (5)	4/174 (2.3)	7/200 (3.5)	0.49
Immunosuppression or autoimmune (5)	0/174 (—)	2/200 (1.0)	0.50
Other chronic conditions^††^ (6)	18/174 (10.3)	23/199 (11.6)	0.71
**Preterm birth (born <37 weeks gestation) (50)**	34/146 (23.3)	38/183 (20.8)	0.58
**Maternal vaccination during pregnancy** ^§§^	28 (15.9)	65 (32.0)	<0.01
Timing of maternal vaccination^¶¶^ (3)			
Early pregnancy (first 20 weeks)	17/165 (10.3)	26/164 (15.9)	0.14
Late pregnancy (21 weeks–14 days before delivery)	9/157 (5.7)	38/176 (21.6)	<0.01
**Maternal vaccine type**			
Pfizer-BioNTech	20 (71.4)	35 (53.9)	0.11
Moderna	8 (28.6)	30 (46.2)	
Behavioral factors***			
Breastfeeding (103)	76/138 (55.1)	90/138 (65.2)	0.09
Child care (108)	6/135 (4.4)	9/136 (6.6)	0.43

**Abbreviation:** SVI = Social Vulnerability Index.

* Infants were enrolled from 20 pediatric hospitals in 17 states. *Northeast*: Boston Children’s Hospital (Massachusetts), Cooperman Barnabas Medical Center (New Jersey), and Children’s Hospital of Philadelphia (Pennsylvania); *Midwest:* Akron Children’s Hospital (Ohio), Nationwide (Ohio), Children’s Mercy Kansas City (Missouri), Mayo Clinic (Minnesota), Riley Children’s (Indiana), Lurie Children’s Hospital (Illinois), Minnesota Masonic (Minnesota), and Children’s Hospital of Michigan (Michigan); *South:* Arkansas Children’s Hospital (Arkansas), University of North Carolina at Chapel Hill Children’s Hospital (North Carolina), Medical University of South Carolina Children’s Health (South Carolina), Texas Children’s Hospital (Texas), Children’s Hospital of New Orleans (Louisiana), and Children’s Healthcare of Atlanta, Emory (Georgia); *West:* Children’s Hospital Colorado (Colorado), Children’s Hospital Los Angeles (California), and University of California San Diego-Rady Children’s Hospital (California).

^†^ If N is less than total.

^§^ Testing for statistical significance was conducted using the Pearson chi-square test and Fisher’s exact test for comparisons with fewer than five observations. Wilcoxon rank-sum tests were used to compare continuous data.

^¶^ CDC/Agency for Toxic Substances and Disease Registry SVI documentation is available at https://www.atsdr.cdc.gov/placeandhealth/svi/index.html. Median SVI for case-infants and control-infants are based on 2018 U.S. SVI data. The SVI ranges from 0 to 1.0, with higher scores indicating greater social vulnerability. One control-infant was missing an SVI score.

** January numbers do not reflect the entire month. Patients included were admitted through January 17, 2022.

^††^ Other chronic conditions included rheumatologic/autoimmune disorder, hematologic disorder, renal or urologic dysfunction, gastrointestinal/hepatic disorder, metabolic or confirmed or suspected genetic disorder, or atopic or allergic condition.

^§§^ COVID-19 vaccination status included the following two categories: 1) unvaccinated (mothers who did not receive COVID-19 vaccine doses before their infant’s hospitalization) or 2) vaccinated (mothers who completed their 2-dose primary mRNA COVID-19 vaccination series during pregnancy and ≥14 days before delivery).

^¶¶^ Timing of vaccination is based on date of receipt of the second dose of a 2-dose primary mRNA COVID-19 vaccine series during pregnancy.

*** Behavioral factors are reported during interview with mother or proxy. Breastfeeding included any breastfeeding (either exclusive or partial).

Among case-infants, 43 (24%) were admitted to an intensive care unit (ICU) (Table 2). A total of 25 (15%) case-infants were critically ill and received life support during hospitalization, including mechanical ventilation, vasoactive infusions, or extracorporeal membrane oxygenation (ECMO); among these critically ill infants, one (0.4%) died. Of the 43 case-infants admitted to an ICU, 88% had mothers who were unvaccinated. The mothers of the one case-infant who required ECMO and one case-infant who died were both unvaccinated.

**TABLE 2:** Clinical outcomes and severity among case-infants aged <6 months hospitalized with COVID-19, by maternal vaccination status during pregnancy* — 20 pediatric hospitals, 17 states,^†^ July 2021–January 2022

Characteristic (no. unknown)	Maternal vaccination status during pregnancy, n/N (%)		
	Total (N = 176)	Unvaccinated (n = 148)	Vaccinated (2-doses of mRNA COVID-19 vaccine) (n = 28)
**Intensive care unit admission**	**43/176 (24.4)**	38/148 (25.7)	5/28 (17.9)
**Critically ill infants on life support (4)**	**25/172 (14.5)**	21/144 (14.6)	4/28 (14.3)
Invasive mechanical ventilation (4)	**11/172 (6.4)**	10/144 (6.9)	1/28 (3.6)
Noninvasive mechanical ventilation (4)	**18/172 (10.5)**	15/144 (10.4)	3/28 (10.7)
Vasoactive infusions (4)	**6/172 (3.5)**	5/144 (3.5)	1/28 (3.6)
Extracorporeal membrane oxygenation (4)	**1/172 (0.6)**	1/144 (0.7)	0/28 (—)
**Infants with discharge data, n/total N (%)**	**170/176 (96.6)**	142/148 (96.0)	28/28 (100)
Hospital length of stay, median days^§^ (IQR) (8)	**2 (1–3)**	2 (1–3)	2 (1–5)
Died before discharge (6)	**1/170 (0.6)**	1/142 (0.7)	0/28 (—)

* COVID-19 vaccination status included the following two categories: 1) unvaccinated (mothers who did not receive COVID-19 vaccine doses before their infant’s hospitalization) or 2) vaccinated (mothers who completed their 2-dose primary mRNA COVID-19 vaccination series during pregnancy and ≥14 days before delivery).

^†^ Infants were enrolled from 20 pediatric hospitals in 17 states. *Northeast*: Boston Children’s Hospital (Massachusetts), Cooperman Barnabas Medical Center (New Jersey), and Children’s Hospital of Philadelphia (Pennsylvania); *Midwest:* Akron Children’s Hospital (Ohio), Nationwide (Ohio), Children’s Mercy Kansas City (Missouri), Mayo Clinic (Minnesota), Riley Children’s (Indiana), Lurie Children’s Hospital (Illinois), Minnesota Masonic (Minnesota), and Children’s Hospital of Michigan (Michigan); *South:* Arkansas Children’s Hospital (Arkansas), University of North Carolina at Chapel Hill Children’s Hospital (North Carolina), Medical University of South Carolina Children’s Health (South Carolina), Texas Children’s Hospital (Texas), Children’s Hospital of New Orleans (Louisiana), and Children’s Healthcare of Atlanta, Emory (Georgia); *West:* Children’s Hospital Colorado (Colorado), Children’s Hospital Los Angeles (California), and University of California San Diego-Rady Children’s Hospital (California).

^§^ Hospital length of stay was missing for eight case-infants born to unvaccinated mothers.

VE of a completed 2-dose maternal primary mRNA COVID-19 vaccination series during pregnancy against COVID-19–associated hospitalization in infants aged <6 months was 61% (95% CI = 31% to 78%) (Table 3). Among 93 mothers classified as vaccinated, 90 (97%) had documented dates of vaccination. Effectiveness of a completed 2-dose COVID-19 vaccination series early in pregnancy (first 20 weeks) was 32% (95% CI = –43% to 68%), although the confidence interval was wide and should be interpreted with caution, and later in pregnancy (21 weeks through 14 days before delivery) was 80% (95% CI = 55% to 91%).

**TABLE 3:** Effectiveness* of maternal 2-dose primary mRNA COVID-19 vaccination against COVID-19-associated hospitalization in infants aged <6 months, by timing of maternal vaccination during pregnancy^†^ — 20 pediatric hospitals, 17 states,^§^ July 2021–January 2022

Timing of maternal vaccination during pregnancy^†^	No. vaccinated^¶^/Total (%)		Vaccine effectiveness,* % (95% CI)
	Case-infants	Control-infants	
Any time	28/176 (15.9)	65/203 (32.0)	61 (31 to 78)
Early (first 20 weeks)	17/165 (10.3)	26/164 (15.9)	32 (–43 to 68)
Late (21 weeks’ gestation through 14 days before delivery)	9/157 (5.7)	38/176 (21.6)	80 (55 to 91)

* Vaccine effectiveness estimates were based on odds of antecedent maternal vaccination during pregnancy in case-infants versus control-infants, adjusted for U.S. Census region, admission date (biweekly intervals), continuous age, sex, and race/ethnicity (non-Hispanic White, non-Hispanic Black, non-Hispanic other, Hispanic of any race, or unknown).

^†^ Timing of vaccination is based on date of receipt of the second dose of a 2-dose primary mRNA COVID-19 vaccination series during pregnancy. Gestational age was missing for seven of 90 (7.8%) infants born to vaccinated mothers with known timing of the second dose, and for these infants classification of vaccination timing was based on gestational age of 40 weeks.

^§^ Infants were enrolled from 20 pediatric hospitals in 17 states. *Northeast*: Boston Children’s Hospital (Massachusetts), Cooperman Barnabas Medical Center (New Jersey), and Children’s Hospital of Philadelphia (Pennsylvania); *Midwest:* Akron Children’s Hospital (Ohio), Nationwide (Ohio), Children’s Mercy Kansas City (Missouri), Mayo Clinic (Minnesota), Riley Children’s (Indiana), Lurie Children’s Hospital (Illinois), Minnesota Masonic (Minnesota), and Children’s Hospital of Michigan (Michigan); *South:* Arkansas Children’s Hospital (Arkansas), University of North Carolina at Chapel Hill Children’s Hospital (North Carolina), Medical University of South Carolina Children’s Health (South Carolina), Texas Children’s Hospital (Texas), Children’s Hospital of New Orleans (Louisiana), and Children’s Healthcare of Atlanta, Emory (Georgia); *West:* Children’s Hospital Colorado (Colorado), Children’s Hospital Los Angeles (California), and University of California San Diego-Rady Children’s Hospital (California).

^¶^ COVID-19 vaccination status included the following two categories: 1) unvaccinated (mothers who did not receive COVID-19 vaccine doses before their infant’s hospitalization) or 2) vaccinated (mothers who completed their 2-dose primary mRNA COVID-19 vaccination series during pregnancy and ≥14 days before delivery).

## Discussion

During July 2021–January 2022, maternal completion of a 2-dose primary mRNA COVID-19 vaccination series during pregnancy was associated with reduced risk for COVID-19 hospitalization among infants aged <6 months in a real-world evaluation at 20 U.S. pediatric hospitals during a period of Delta and Omicron variant circulation. Among 176 infants aged <6 months hospitalized with COVID-19, 148 (84%) were born to mothers who were not vaccinated during pregnancy. Although booster doses are recommended for pregnant women, VE of maternal booster doses received during pregnancy could not be assessed because of small sample size, which likely underestimated VE. Overall, these findings indicate that maternal vaccination during pregnancy might help protect against COVID-19 hospitalization among infants aged <6 months.

COVID-19 during pregnancy is associated with severe illness and death (*7*), and pregnant women with COVID-19 are more likely to experience preterm birth, stillbirth, and other pregnancy complications (*8*). Vaccination is recommended for pregnant women to prevent COVID-19, including severe illness and death. COVID-19 vaccination is safe and effective when administered during pregnancy (*9*,*10*). Receipt of COVID-19 vaccination during pregnancy is associated with detectable maternal antibodies in maternal sera at delivery, breast milk, and infant sera indicating transfer of maternal antibodies (*3*–*5*). The higher VE point estimates among infants born to women vaccinated later in pregnancy are consistent with the possibility of transplacental transfer of SARS-CoV-2–specific antibodies that might provide protection to infants. The optimal timing of maternal vaccination for the transfer of antibodies to protect the infant is currently uncertain, and the direct effect of maternal COVID-19 vaccination in preventing severe COVID-19 in infants has not previously been described. Further, with infants not currently age-eligible for vaccination and infant hospitalization rates remaining at the highest levels of the pandemic,*** this study suggests that maternal COVID-19 vaccination during pregnancy might protect infants aged <6 months from COVID-19–related hospitalization.

The findings in this report are subject to at least seven limitations. First, VE could not be assessed directly against specific variants. Second, the sample was too small to assess VE by pregnancy trimester of vaccination, and the small sample size resulted in wide confidence intervals for some estimates that should be interpreted with caution. Third, the analysis did not assess whether pregnant women were infected with SARS-CoV-2 before or during pregnancy, which might have provided maternal antibodies. Fourth, residual confounding such as additional differences in behaviors between vaccinated and unvaccinated mothers, including whether mothers had prenatal care, that might affect risk for infection cannot be excluded, and potential confounders (e.g., breastfeeding, child care attendance, and prematurity) could not be accounted for in the model because this information was not available for all infants. Fifth, because this analysis included self-reported data for a few participants, maternal vaccination status might be misclassified for a few infants, or there might be imperfect recollection of whether the mother completed COVID-19 vaccination during pregnancy. Sixth, immunocompromising maternal conditions were not collected to determine whether mothers needed an additional mRNA COVID-19 vaccine dose to complete their primary series. Finally, VE of maternal booster doses received during pregnancy could not be assessed because of small sample size.

Completion of a 2-dose primary mRNA COVID-19 vaccination series during pregnancy was associated with reduced risk for COVID-19–associated hospitalization among infants aged <6 months, and protection was higher among infants whose mothers were vaccinated later in pregnancy. Additional evaluation should examine timing of vaccination before pregnancy compared with during pregnancy. CDC recommends that women who are pregnant, are breastfeeding, are trying to get pregnant now, or might become pregnant in the future get vaccinated and stay up to date with COVID-19 vaccination.^†††^

**Summary**

**What is already known about this topic?**

COVID-19 vaccination during pregnancy is recommended to prevent severe illness and death in pregnant women. Infants are at risk for COVID-19–associated complications, including respiratory failure and other life-threatening complications.

**What is added by this report?**

Effectiveness of maternal completion of a 2-dose primary mRNA COVID-19 vaccination series during pregnancy against COVID-19 hospitalization among infants aged <6 months was 61% (95% CI = 31% to 78%). Effectiveness of completion of the primary COVID-19 vaccine series early and later in pregnancy was 32% (95% CI = –43% to 68%) and 80% (95% CI = 55% to 91%), respectively.

**What are the implications for public health practice?**

Completion of a 2-dose mRNA COVID-19 vaccination series during pregnancy might help prevent COVID-19 hospitalization among infants aged <6 months.

## Footnotes

* These authors contributed equally to this report.

† These senior authors contributed equally to this report.

§ https://www.cdc.gov/coronavirus/2019-ncov/vaccines/recommendations/pregnancy.html.

¶
https://covid.cdc.gov/covid-data-tracker/#variant-proportions

** Symptomatic COVID-19–like illness was defined as one or more of the following: fever, cough, shortness of breath, gastrointestinal symptoms (e.g., diarrhea, vomiting, or “stomachache”), use of respiratory support (high-flow oxygen by nasal cannula, new invasive or noninvasive ventilation) for the acute illness, or new pulmonary findings on chest imaging consistent with pneumonia. Four case-infants tested at an outside hospital or other facility had some missing data on positive test results and were not retested at the study hospital.

†† Mothers were defined as vaccinated after completing their 2-dose primary mRNA COVID-19 vaccine series during pregnancy, including both doses received during pregnancy or the first dose received before pregnancy and the second dose, completing their primary series, received during pregnancy. Data on maternal moderately or severely immunocompromising conditions were not recorded for mothers of enrolled infants to determine whether mothers needed an additional mRNA COVID-19 vaccine dose to complete their primary series.

§§ 45 C.F.R. part 46.102(l)(2), 21 C.F.R. part 56; 42 U.S.C. Sect 241(d); 5 U.S.C. Sect 552a; 44 U.S.C. Sect 3501 et seq.

¶¶ Other reasons for excluding infants from the analysis included May or June hospital admission (two); birth to mothers who received Janssen (Johnson & Johnson) COVID-19 vaccine (four), who received their second dose of vaccine <14 days before delivery (three), who received a 2-dose primary mRNA COVID-19 vaccine before pregnancy (seven), or with unknown vaccination status (one); infants who received a positive SARS-CoV-2 test result but were admitted for non–COVID-19 reasons (four); and SARS-CoV-2 testing >10 days after illness onset or >3 days from hospitalization (two).

***
https://gis.cdc.gov/grasp/covidnet/COVID19_5.html

†††
https://emergency.cdc.gov/han/2021/han00453.asp
